# Shared reproductive disruption, not neural crest or tameness, explains the domestication syndrome

**DOI:** 10.1098/rspb.2022.2464

**Published:** 2023-03-29

**Authors:** Ben Thomas Gleeson, Laura A. B. Wilson

**Affiliations:** ^1^ Fenner School of Environment and Society, The Australian National University, Acton, Australian Capital Territory 2601, Australia; ^2^ School of Archaeology and Anthropology, The Australian National University, Acton, Australian Capital Territory 2601, Australia; ^3^ School of Biological, Earth and Environmental Sciences, University of New South Wales, Kensington, Sydney, New South Wales 2052, Australia

**Keywords:** animal domestication, neural crest cell hypothesis, unconscious selection, island effect, self-domestication

## Abstract

Altered neural crest cell (NCC) behaviour is an increasingly cited explanation for the domestication syndrome in animals. However, recent authors have questioned this explanation, while others cast doubt on whether domestication syndrome even exists. Here, we review published literature concerning this syndrome and the NCC hypothesis, together with recent critiques of both. We synthesize these contributions and propose a novel interpretation, arguing shared trait changes under ancient domestication resulted primarily from shared disruption of wild reproductive regimes. We detail four primary selective pathways for ‘reproductive disruption' under domestication and contrast these succinct and demonstrable mechanisms with cryptic genetic associations posited by the NCC hypothesis. In support of our perspective, we illustrate numerous important ways in which NCCs contribute to vertebrate reproductive phenotypes, and argue it is not surprising that features derived from these cells would be coincidentally altered under major selective regime changes, as occur in domestication. We then illustrate several pertinent examples of Darwin's ‘unconscious selection' in action, and compare applied selection and phenotypic responses in each case. Lastly, we explore the ramifications of reproductive disruption for wider evolutionary discourse, including links to wild ‘self-domestication' and ‘island effect’, and discuss outstanding questions.

## 1. Introduction

Altered embryonic neural crest cell (NCC) behaviour is a widely cited explanation for the domestication syndrome [1], a suite of shared, apparently associated, changes, observed in domesticated populations when compared to their wild ancestors or relatives. These changes are thought to have emerged spontaneously during the earliest stages of animal domestication, thus do not result from deliberate, or ‘conscious', selection by human domesticators. Vertebrate NCCs are a pluripotent lineage of embryonic cells which give rise to a wide range of tissues, organs and structures in all vertebrates [2–4]. They have been claimed as a ‘common denominator' which explains domestication syndrome traits as a form of ‘mild neurocristopathy' due to pleiotropic genetic disruption of widespread NCC contributions to the wild phenotype [1,5]. Despite its increasing prominence, however, several authors have recently questioned the NCC explanation for domestication syndrome [6,7], while others prominently argue there is little evidence that domestication syndrome even exists [8–10].

**Figure 1.**
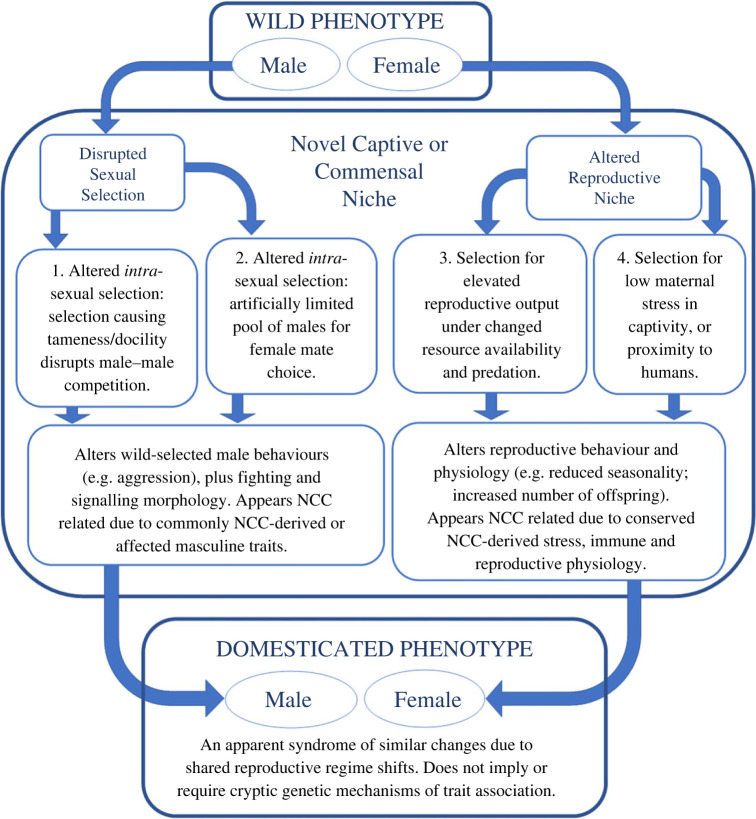
Four primary forms of shared reproductive disruption likely to promote shared trait changes of domestication syndrome across different taxa. Notes: NCC, neural crest cell.

Here, we succinctly assess and synthesize key aspects of agreement and difference between these, and other, scientific perspectives of the domestication syndrome and its causes. Based on this synthesis, we propose a novel interpretation, arguing there *is* a collection of common, if notably variable, trait changes which may be referred to as ‘domestication syndrome', but that these shared phenotypic shifts primarily result from shared disruption of pre-existing wild reproductive regimes. In essence, we observe that wild phenotypes experiencing functionally similar changes in selective regimes should often show functionally similar phenotypic responses. We emphasize changes in four primary selective pathways (figure 1); being: (1) disrupted inter-sexual and (2) intra-sexual selection in males, and (3) changed resource availability and predation pressure, plus (4) intensified maternal stress, affecting reproductive physiology in females. This expanded range of predictably altered selective influences could include, but is not limited to, widely cited ‘selection for tameness' (discussed in further detail below). We note these are not the only selective shifts impacting domesticated phenotypes, but suggest they sufficiently encompass the most influential shared selective factors likely to promote common trait changes which comprise the domestication syndrome.

A logical implication of our selection-focused hypothesis is that shared domestication syndrome traits do not require any shared genetic or biological mechanisms of association. In turn, this suggests commonly observed trait changes only coincidentally occur in features and physiology derived from vertebrate NCCs. From our perspective, this seems a reasonable supposition since, as we document below, these pluripotent cells contribute to an astonishing range of important, conserved and derived, reproductively relevant traits, features, and systems across both sexes of all vertebrates [11,12]. Given their widespread and significant phenotypic contributions, it should not be surprising that NCC-related features are often altered under major selective regime shifts, as typically experienced during domestication.

In summary, we argue domestication syndrome is most accurately considered as a variable, though overlapping, response to shared disruption of common sexual and reproductive selective regimes. Notably, this selective trait association—occurring primarily via four pathways of reproductive disruption (figure 1)—affords an extended explanatory power beyond traditional domestication research. For example, from this perspective, recently posited cases of ‘self-domestication' (e.g. on islands; in commensal and urban wild populations; among panins and other primates; and in ancient hominin evolution) would also likely result from selective regime shifts affecting reproductively relevant behaviour, physiology and morphology in similar ways across different populations and taxa in each context. These wild trait changes might also primarily arise in NCC-derived features, but, we argue, this is due to the ubiquity and reproductive relevance of these derivatives. In effect, our selectively focused hypothesis can succinctly explain the emergence of similar evolutionary changes across a wide range of contexts—both domesticated and wild—without invoking selection for tameness, or cryptically shared genetic mechanisms of pleiotropic trait association.

## 2. A history of domestication syndrome

Researchers have long discussed a suite of apparently associated and shared trait changes seen in different domesticated animal populations when compared to their wild ancestors or relatives. This phenomenon has been referred to in various ways after Darwin [13,14] first noted the ‘correlated variation' of certain traits in domesticated animals. Later notable authors have referred to certain ‘generalities' across the domesticates [15], or to ‘general effects' of domestication [16]. Groves [17] employed the phrase ‘parallel changes', whereas Belyaev [18]—following Vavilov [19]—discussed ‘homologous variability'. Jensen [20] invoked a singular ‘domestication phenotype' to encapsulate five important trends shared by most domesticated species. By contrast, Price [21,22], Kohane & Parsons [23] and Zeder [24] all employ ‘domestic phenotype', but only to differentiate from the wild form of a given population; thus not in reference to a suite of traits shared by different taxa.

The phrase ‘domestication syndrome' was first developed, and has long been used, in plant domestication literature [1,5]. Its application followed Harlan *et al*.'s [25] original 1973 description of an ‘adaptation syndrome' in domesticated grains, caused by unintended selective effects from ancient human cultivation. In 1984, Hammer [26]—citing Faegri [27]—employed the term ‘domestication syndrome' primarily to discuss Harlan *et al*.'s [25] concept in crop plants, but also suggested it could be applied to domesticated animals due to certain ‘parallels' in each case. While noting its origins in plant domestication literature, Wilkins *et al*.'s [1] initial proposal of the NCC hypothesis used ‘domestication syndrome' explicitly to describe the shared suite of changes previously noted in domesticated animals. Use of this term in relation to animals has increased substantially following their contribution [5].

According to Harlan *et al*. [25] crop ‘adaptation syndrome' emerged across multiple domesticated grains as a result of ‘automatic selection' due to humans replanting previously harvested seed. This description of ‘automatic' selective effects closely resembles Darwin's [13,14] earlier concept of ‘unconscious selection', which he used to describe effects from human actions that were not intended to produce heritable changes within a given population. Darwin [13,14] originally theorized two important phases of animal domestication: one involving ‘unconscious', and the other ‘conscious', selection by humans. He reasoned the unconscious phase began when ancient animal populations first entered captivity or commensalism, and arose due to unintended selective effects from novel association with humanity. Later, as humans came to recognize the potential for deliberate selection of certain traits within a population, there began a more conscious phase of intentional, or ‘methodical', selection; culminating in targeted breeding practices used to maintain desired traits and formally designated breeds of many species [13,14].

From a genetic perspective, researchers often distinguish between ‘domestication traits' and ‘improvement traits'; the former arising from unconscious selection during the initial phases of domestication, and the latter from conscious selection, often only in a sub-population of domesticates, such as individual breeds [28]. Crucially, domestication syndrome involves a shared suite of unintended ‘domestication traits' that emerged, in apparent association, during the ancient unconscious phases of each animal's pathway to domestication. Despite this, as recently noted [7], some traits commonly attributed to domestication syndrome are, in fact, breed specific, which implies relatively *conscious* trait selection by humans, and should exclude them from domestication syndrome. This is because, where there is conscious human selection for a given trait, this activity alone provides an obvious cause for its appearance in a population, and thus precludes any need of further explanation.

Several previous reviews have surveyed and concisely summarized the range of traits and features attributed to domestication syndrome in animals (e.g. [1,8,29]). Increased ‘tameness', or ‘docility', is widely acknowledged as the most consistently observed change relative to the wild state; however, decreased brain and body size, and spontaneously altered pigmentation are also relatively ubiquitous [1,8,17,24,29–31]. Other common traits include: shortened muzzle/crania/palate; reduced tooth sizes; changes in the number of vertebrae; and shifts in oestrus cycling or reproductive output [1,29]. Notably, however, while domestication syndrome is commonly defined by shared trait changes, it is also acknowledged as highly variable; in fact, no two domesticates show the exact same range of altered traits [5,8,29].

Relatively ubiquitous ‘tame' or ‘docile' animal behaviour has been described in several ways by different authors. Wilkins *et al*. [1] highlight dampened ‘aggression' and ‘fearfulness'. Similarly, Agnvall *et al*. [32] and Albert *et al*. [33] discuss ‘reduced fear of humans' and a ‘lack of aggressive and defensive reactions towards humans', respectively. In a broader sense, Kohane & Parsons [23] identified reduced behavioural responses to general environmental ‘stress'. These shifts towards relative ‘tameness', however defined, are widely thought to arise from changes in domesticate nervous systems and neuroendocrine function [1,33,34]. Other authors emphasize altered brain morphology; especially diminished limbic systems, either as part of overall brain size reduction [30,35], or as relative shifts in different brain areas, as noted in rabbits [36]. Increased social affiliation has also been proposed to dampen domesticate aggression, especially via altered oxytocin regulation [37,38]. Lastly, Hemmer [39] depicted domestication as a general ‘decline of environmental appreciation', and thus considered various physical adaptations (e.g. poorer hearing and eyesight) which might also reduce animal perception of potential environmental stressors.

A common observation regarding shared traits of domestication syndrome is that these changes emerge in apparent association across the affected populations. The long-running Russian farmed fox experiment [18], which applied controlled breeding selection for tame behaviour to a captive population of silver foxes, is probably the most famous demonstration of these associations. In this experiment, researchers repeatedly inserted a gloved hand into animal cages and recorded levels of fear and aggression displayed. Three distinct populations of foxes—designated as ‘tame', ‘aggressive' and ‘control' lineages—were developed based on their observed behavioural responses [18,40,41]. Within a few generations, the tamest foxes were reported to seek human contact without fear, but had also acquired multiple *unselected* features typical of traditional domesticates, including relatively shortened jaws, drooping ears, piebald pigmentation, reduced stress physiology and altered reproductive timing [18,40,42]. Over a period of decades, this experimental demonstration of associated changes in response to ‘selection for tameness' has become a key pillar of scientific and popular discourse regarding domestication and domestication syndrome [1,18,20,43,44].

Animal domestication syndrome has an intrinsic scientific appeal, in part, because it appears to offer an objective, measurable and biologically based indication of the domesticated state. Archaeologists, in particular, have long debated appropriate methods for differentiating wild and domesticated animal remains in ancient deposits [45,46]. Beyond study of traditional domesticates, however, domestication syndrome has also been used to support novel perspectives in wider evolutionary research via an expanding discourse regarding wild ‘self-domestication', including in bonobos [47], in marmosets [48], in early wolf/dog domestication [49], under ‘island effect' [29,50], in commensal mice [51], in urbanized wild foxes [52] and during human evolution [53–57]. These studies are based on observation of domestication-like traits (in effect, domestication syndrome) in populations and taxa not previously considered as subject to any form of domestication.

## 3. The neural crest hypothesis

Given the apparent association of shared behavioural, morphological and physiological changes across different domesticated populations and taxa, multiple previous authors have hypothesized some commonly shared explanation. Proposed mechanisms have included: shared heterochronic changes, or ‘systemic neoteny' [58]; widespread transition towards an ‘r-selected' life history [59]; shared shifts in thyroid hormone circulation [60,61]; as well as altered androgens, or androgen receptors [62]. Having noted that domestication syndrome appears to affect many traits or features ultimately derived from neural crest cells (NCCs), in 2014, Wilkins *et al*. [1] proposed the novel hypothesis that domestication syndrome in animals results from shared disruption of NCC regulatory genetics influencing the migration, or proliferation, of these cells during embryonic development. These authors have described NCCs as a ‘common denominator' which might pleiotropically link the observed traits as symptoms of a ‘mild neurocristopathy' [1,5]—a suite of pathologies involving associated developmental abnormalities, derived from NCC regulatory defects [11].

Vertebrate NCCs are a pluripotent lineage of early embryonic cells, which initially line the two crests of the neural fold, fusing them to create the neural tube, then dispersing throughout the developing embryo to form numerous other types of cells, tissues, structures and organs [2–4]. They are a defining feature of vertebrate evolution, and have been described as ‘a fourth germ layer' in vertebrate development [3]. Wilkins *et al*. [1,5] suggest that ancient selection for tame behaviour—like that applied in the Russian fox experiment—caused heritable reductions in aggression by disrupting documented NCC contributions to the vertebrate endocrine and autonomic nervous systems [11,63]; especially via the pituitary, adrenal medulla, and related ganglia. They theorize that because the applied behavioural selection alters NCC behaviour in a given lineage or population, other NCC-derived features are pleiotropically affected, provoking a wider range of associated shifts (i.e. ‘domestication syndrome') as an unintended side-effect [1,5]. Since its proposal, the NCC hypothesis has been widely cited, and credited as the ‘most popular' [7] explanation for the shared traits of domestication syndrome.

In a related article, Wilkins [64] directly compared the NCC hypothesis to Crockford's [60,61,65] earlier proposal that shared changes under domestication were caused by altered thyroid hormone regimes. In this, he argued changed NCC regulation was a more compelling explanation primarily based on evidence of altered NCC genetics in domesticated populations [64]. In their more recent paper, Wilkins *et al*. [5] also acknowledged support for the NCC hypothesis claimed by studies showing evidence of past selection affecting various neural system genes in different domesticated lineages [66,67]. In fact, multiple studies have noted NCC-related changes in specific taxa, including dogs [67], cats [68], horses [66,69], dromedaries and Bactrian camel [70], rabbits [71], archaic humans [72] and *Homo sapiens* [73]. In contrast to claims of genetic support for the NCC hypothesis, however, evidence from morphological research remains equivocal [74] and its exploration to date has been limited; in part due to a lack of mechanistic frameworks to guide hypothesis testing.

## 4. Recent criticism and response

Despite apparent genetic support for the NCC hypothesis, based on their review of related literature, Lord *et al*. [8,10] recently argued there is little evidence that domestication syndrome actually exists. As part of their discussion, they criticized Belyaev's [18] farmed fox experiment, noting its founding population was drawn from a pre-existing fox-farm, rather than from the wild, and that features attributed to domestication syndrome were already present in these founders, thus had not emerged *de novo* due to experimental ‘selection for tameness' [8]. Elsewhere, they highlighted that the experiment had been established using a small number of individuals chosen for unusually friendly behaviour, making a strong founder effect highly likely [9]. In addition, they claim their own review of other literature had failed to find ‘a single species' with published evidence meeting their formal criteria for the domestication syndrome [8].

In direct response, several subsequent contributions defended the existence of domestication syndrome, and the rigour (and findings) of the Russian fox experiment (e.g. [75,76]). Perhaps most succinctly, Zeder [76] argued *evidence of domestication syndrome* in founding foxes logically cannot be used to refute the existence of domestication syndrome. Wright *et al*. [7] also noted that Lord *et al*.'s [8] review adopted hypothetical assumptions drawn from the Russian fox experiment, combined with expectations of cross-species pleiotropic association of traits—as implied by the NCC hypothesis. They argue this led to an arbitrarily narrow definition of domestication syndrome, which Lord *et al*. [8] explicitly state is, ‘a suite of traits that rises in frequency as a direct consequence of selection on tameness due to linkage or pleiotropy'. In effect, Lord *et al*. [8] assumed that, to provide evidence for domestication syndrome, any observed feature must necessarily appear in response to overt ‘selection on tameness', and must arise in concert with other symptoms specifically due to gene-based mechanisms of trait association.

According to Wright *et al*. [7], these assumptions meant Lord *et al*. [8] excluded many relevant observations from their review, thus precluding a comprehensive assessment of domestication syndrome, and limiting the relevance of their critique to two causal hypotheses: specifically, selection for tameness, and genetic pleiotropy. By contrast, Wright *et al*. [7] argued a narrow suite of shared changes does appear commonly across different domesticates; but—*contra* Wilkins *et al*. [1]—they suggest these similarities are unlikely to be associated via shared shifts in NCC regulatory genetics. These same authors reiterate their position more extensively in Johnsson *et al*. [6], where they outlined three main critiques, which we summarize here as: (1) trait variability: meaning the domestication syndrome does not present as universal changes in NCC-derived features; (2) shared traits need not require a shared genetic mechanism; (3) even if there was a shared genetic mechanism, evidence that it would be NCC-related is weak. They summarized their assessment by suggesting the NCC hypothesis is an implausible ‘explanation looking for a problem' [6].

In defence of the NCC hypothesis, Wilkins *et al*. [5] discussed apparent misrepresentations in Johnsson *et al*. [6], and emphasized the demonstrable promotion of scientific interest and activity, especially via support from multiple findings of past selection on NCC candidate-genes in ancient domesticates (e.g. [66,67,77]). Despite this, however, Johnsson *et al*. [6] had argued evidence of past selection on genes associated with NCCs is not necessarily evidence for the specific pleiotropic mechanisms implied by the NCC hypothesis to explain domestication syndrome. At best, they suggest, such evidence shows selection on undefined NCC-derived features—though it may also reflect change in developmental regulation of non-NCC-derived phenotypic components [6]. In further defence of the NCC hypothesis, Wilkins *et al*. [5] also invoked its falsifiability; effectively suggesting it could be falsified: (1) if ablation of embryonic NCCs led to no expected changes in NCC-derived features; (2) if there was no significant reduction in NCC counts between domesticates and wild comparators; or (3) if no NCC gene mutations were apparent in domesticate comparators.

## 5. The ‘reproductive disruption' hypothesis

Having examined existing literature on domestication syndrome and the NCC hypothesis, here we synthesize key findings and reiterate our own explanatory proposal. As an initial basis for our views, we accept published evidence that the domestication syndrome does exist, however, we acknowledge it is a relatively variable condition, and recognize several observations suggesting it cannot be adequately explained by selection for tameness triggering genetically associated changes via altered NCC behaviour [6–8]. Following Zeder [76], we note that the pre-existence of domestication syndrome traits in Russian experimental foxes in no way undermines the existence of domestication syndrome. However, as Lord *et al*.'s [8,9] contributions highlight, this pre-existence must logically weaken claims that Belyaev's experimental ‘selection for tameness' was the catalyst for their emergence (see §7).

As such, we agree with contributions from Wright *et al*. [7], Zeder [76], Trut *et al*. [75], Wilkins *et al*. [1,5] and many others (e.g. [15–17,20,31,39]), that a variable collection of common trait changes does appear relatively reliably in different domesticated taxa. However, we also concur with Lord *et al*.'s [8] assertion that understanding this phenomenon requires ‘a more comprehensive approach focused on essential adaptations to human-modified environments'. Further, we appreciate and accept Wilkins *et al*.'s [1,5] key observation, that many traits of domestication involve changes in NCC-derived features—an insight which has sparked much further study. However, we agree with Wright *et al*. [7] and Johnsson *et al*. [6] that NCC-related pleiotropy is unlikely to explain why these shared and associated traits emerge across the different populations. From this synthesis position, we offer an alternative explanatory framework, positing an expanded range of shared selective influences (figure 1) which should tend to alter wild phenotypes in similar ways due to pre-existing commonalities in reproductive strategies and evolved physiological constraints.

In essence, we hypothesize that the variable suite of traits comprising the domestication syndrome emerges repeatedly in different taxa because shared disruption of wild selective regimes often prompts similarly shared phenotypic responses. Further, we posit that these selective changes alter traits differently in males and females (figure 1) due to sexually differentiated reproductive strategies and physiology. Specifically, we highlight that domestication reliably shifts wild male intra- and inter-sexual selection by subverting natural dominance contest—dampening male conflict and aggression—and restricting the pool of males available for female choice—thus disrupting other modes of male competition. In addition, domestication promotes common changes in female reproductive physiology—e.g. as loss of seasonality and reduced maternal stress—due to selection (natural and artificial) for elevated reproduction under (1) changed predation pressure and resource availability, and (2) elevated stress due to novel captivity, or proximity to humans. Since reproductive features are consistently sexually, and often phylogenetically, specific, our selectively focused hypothesis succinctly addresses why the shared suite of traits attributed to domestication syndrome will typically vary by sex and taxon.

We recognize we are not the first authors to propose a selective explanation for similar phenotypic shifts seen under domestication (e.g. [18,31,59,78]). However, we feel our contribution is novel for several reasons, including that: (i) it cogently integrates and resolves multiple diverse perspectives from recent domestication debate; (ii) it addresses the domestication syndrome directly and in its entirety; (iii) it explicitly eschews cryptic genetic trait association, in favour of shared selective shifts; (iv) it highlights four primary, sexually dimorphic, pathways of selective regime change (figure 1); and, in doing so, (v) it can explain why examples of wild ‘self-domestication' (including purported ‘island effect') might often resemble changes commonly seen in traditional animal domesticates.

## 6. An alternative view of NCC changes

To reiterate, we propose that shared phenotypic changes comprising animal domestication syndrome emerge due to multiple shared selective shifts occurring under domestication (figure 1). However, we also accept Wilkins *et al*.'s [1,5] observation that these changes often involve NCC-derived traits and features. As such, in association with our causal hypothesis, we feel obliged to provide an alternative explanation for why domestication commonly alters NCC-derived features and physiology. Briefly, we argue this is simply due to the high proportion of reproductively relevant vertebrate features which derive from the neural crest. These provide regular targets for natural and sexual selection, and are common catalysts of taxonomic differentiation and speciation; whether via male adaptations for fighting and signalling [53], or a range of other pathways [79]. As such, any significant selective regime change (under domestication, or other ecological circumstances) should predictably, but only coincidentally, often affect traits and features derived from these vertebrate cells, whose contributions continue to be elaborated by science [11]. NCCs are involved in multiple phylogenetically primitive physiological functions, and give rise to numerous highly derived reproductive features; including conserved mediators of maternal stress and fecundity in females, and an astonishing variety of adaptations for fighting and signalling in males (table 1).

**Table 1.** Neural crest cell (NCC) relevance to vertebrate reproductive traits and features; especially male secondary sexual features and female reproductive physiology—adapted from Gleeson [53]. HPA, hypothalamic–pituitary–adrenal; HPG, hypothalamic–pituitary–gonadal.

reproductive features		taxonomic examples	role of NCCs
male secondary sexual traits	behaviours	elevated male competition and aggression in multiple taxa	NCCs form the adrenal medulla [80,81] and pituitary [63], affecting autonomic response and androgens via HPA and HPG axes
	craniofacial morphology	craniofacial sexual dimorphisms in multiple taxa, e.g. primate brow ridges, sagittal crests and cheek flanges	most of the vertebrate craniofacial region derived from NCCs [81–84]
	horns, antlers and other headgear	horns, pronghorns, antlers and ossicones in ungulates	NCCs form antlers [85,86] and dermis from which horns, pronghorns, and ossicones emerge, plus frontal bones to which they attach [4,81]
	larger teeth	tooth sexual dimorphism (especially canines), common in multiple taxa	NCCs provide progenitors for tooth odontoblasts and papillae [4,80,81]
	pigmented and structural colorations	pigmented and structurally derived male coloration, in multiple taxa	NCCs provide chromatophores and cellular iridescence in skin, hair, and feathers [4,87]
	vocal signalling	masculine vocalizations, in multiple taxa	NCCs form cartilage of hyoid and larynx [88], plus associated neck and throat muscles [89]
female reproductive physiology	maternal stress	nervous and neuroendocrine systems in multiple taxa	NCCs crucial to adrenal medulla [80,81], pituitary [63] and ganglia of the autonomic nervous system [11]
	reproductive functions	contribute to the HPG axis in multiple taxa	NCC-derived pituitary [63] governs luteinizing and follicle stimulating hormones [90]
	immune functions	immune system and metabolism in multiple taxa	NCC contributions to immune function via the thymus [91], spleen [92], bone marrow [93] and thyroid [94]

Because vertebrate male adaptations for fighting and signalling commonly involve NCC progenitors, predicted change in intra- and inter-sexual selection affecting male domesticates (figure 1) should often alter NCC-derived male features, or sexual dimorphisms governed by NCC-derived physiology (e.g. pituitary functions). In addition, because these masculine traits and sexual dimorphisms vary by taxa, the reproductive disruption hypothesis succinctly accounts for interspecific variability of male trait changes under domestication syndrome. For example, size reduction or absence, of (NCC-derived) horns occurs in taxa where horns are part of male-male competition and signalling. Whereas changes in vocalizations and pigmentation should be more likely where wild males compete to secure reproductive partners via these specific modes of signalling. Reductions in other sexual dimorphisms (e.g. body size, tooth size, cranial shape) will probably occur where these features were previously sexually selected, or are caused by sexually dimorphic physiology.

Shared changes in female reproductive physiology are also likely under domestication, reflecting common selective release from wild reproductive constraints (e.g. food availability and predation), and—in early stages of domestication—elevated maternal stress from novel captivity or commensalism. Stress in captivity, or proximity to humans, negatively affects maternal reproduction in placental mammals due to documented effects on offspring health, development, and mortality [95–97]. Notably, however, female vertebrate reproduction and stress physiology depend on several highly conserved NCC-derived features (table 1), including important components of the HPA and HPG axis, as well as NCC-derived ganglia of the autonomic nervous system. As a result, common female adaptations under a domestic selective environment will probably affect similar NCC-derived stress, immunity, and reproductive features, across different domesticated species. As such, female trait changes under domestication should also often involve NCC-derived features, but this does not require, or imply, NCC-related trait association, or genetic pleiotropy, as a cause of these shared changes. By contrast, we argue, these adaptations are associated by similar selective changes, and occur commonly in NCC-derived features due to their widespread contributions to conserved vertebrate reproductive systems and features.

In summary, the reproductive disruption hypothesis suggests shared traits in multiple domesticates can be explained by shared selective shifts, rather than shared pleiotropic genetic mechanisms. As a result, acknowledged changes in NCC-related features are effectively coincidental to our causal explanation for the domestication syndrome. We argue common change in NCC-derived features is predictable simply because a large proportion of every vertebrate phenotype is derived from this primitive cellular lineage—including an even larger proportion of reproductively relevant features in each sex (table 1). In effect, NCC-derived features regularly provide the phenotypic variability upon which natural and sexual selection operates; under domestication or anywhere else. We are not suggesting pleiotropic mutations resembling ‘mild neurocristopathies' do not occur under domestication, but such effects (e.g. piebald pigmentation) are just one form of the many shared changes that occur. For further clarity, we compare key expectations and implications of the reproductive disruption and NCC [1,5] hypotheses in table 2.

**Table 2.** A comparison of the neural crest cell (NCC) and ‘reproductive disruption’ hypotheses.

hypotheses	posited driver	affects …	via …	leads to …	implies …
NCC hypothesis	selection for tame behaviour	NCC-derived behavioural systems	dampened NCC migration, or proliferation	retarded ontogeny of NCC-derived features: or ‘mild neurocristopathy'	genetic trait association; repeated hypoplasia in NCC-derived features
reproductive disruption	similar changes in selective niche	male sexual traits and female reproductive physiology	standard evolutionary processes of genetic mutation with selection	similar shifts in reproductive behaviour, physiology and morphology	selective trait association; similar variations in reproductively relevant traits

## 7. Illustrating unconscious selection under domestication

As a common example of ancient *unconscious* selection in animals, several authors have theorized that routine culling of dangerous or aggressive males in captive populations (e.g. of pigs, sheep, goats or cattle) must have had automatic, though unintended, effects on those populations over time [24,30,78,98,99]. Given heritable physiological aspects of animal behaviour, culling relatively aggressive individuals should promote docility in subsequent generations. As such, this relatively automatic selection—arising from a natural human intolerance of dangerous or aggressive animals—would lead to tamer domesticated populations over time, even without a *conscious* selective intent among human domesticators.

Other forms of unconscious selection can also occur via influences from the domestic environment more generally. As mentioned, capture and captivity reliably heighten stress levels in most wild animals, and elevated stress constitutes a significant hurdle for wild animal domestication. Females typically experience more stress due to sex-hormone effects upon developing neuroendocrine systems [100,101] and maternal stress leads to poor reproductive outcomes, including: fewer offspring, lower birth weights, and poor health and survivorship of young [95–97]. Given natural variability in stress response, certain behavioural ‘pre-adaptations' [17,24] for domesticated living might enhance the relative fitness of some lineages, or taxa. However, initially poor reproductive capacity has often been overcome, as demonstrated by experimental domestication of wild-caught gray Norway rats, documented by King & Donaldson [102] over 25 generations. According to King ([103], p. 26), the initial captivity of wild rats,

affected reproductive processes adversely, causing sterility in some females and greatly reducing fertility in others. Only six of the [20] wild females bred in captivity, and the litters they cast were small. … wild females, with one exception, seemed incapable of suckling their offspring, and their litters were either destroyed soon after birth or neglected.

Behaviour of these rats suggests high stress levels, which persisted for several generations, even among subsequently captive-born rats. Again, according to King ([103], p. 50),

A high nervous tension and extreme fear of man was shown by all rats in early generations. They ran wildly about the cage … and constantly gnawed the wire netting and other parts of the cage in their efforts to escape from confinement.

Despite their obvious stress, and near complete failure to reproduce, some litters from the captured wild mothers were suckled by pre-domesticated albino foster mothers to ensure future stock. Over subsequent generations, the population showed gradual improvement in female reproductive capacity, until even exceeding the fecundity of rats in the wild [102,103]. There was steady increase in the number of litters per female; in pup birth weights; and in length of female reproductive life [103]. Eventually, all captive-born females were reliably fertile. Besides changed fertility, however, mutations in hair pigment and structure also arose throughout the experiment. These led to various fur and eye colour morphs; with recognized patterns including, hooded, piebald and albino [103]. There was also a ‘stub-tailed' mutation, caused by reduction in the number of tail vertebrae [103]. Notably, this range of changes resembles previously recognized features of domestication syndrome [1,8,29,104]. Over time, significant behavioural changes were also strongly apparent. For example, all rats from later generations

lost their fear of man and were so well adjusted to their new environment that restriction to reproduction induced by removal from their natural habitat had disappeared. ([103], p. 32)

What is crucial, however, is that these behavioural changes were never consciously selected for by the experimenters; as stated by King ([103], p. 51),

As this investigation was designed to study the effects of captivity on gray rats, no attempts were made to tame any of the rats used in this work so that they could be handled as are the rats of various strains maintained for general laboratory purposes.

This absence of deliberate selection for tameness strikingly contrasts with methods applied during the Russian fox experiment [18,40,41]—and, notably, would exclude King & Donaldson's [102] rats from Lord *et al*.'s [8] critical review of domestication syndrome, despite apparent relevance to this topic. In further contrast, however, King & Donaldson's [102] experiment also began with wild-caught rats, whereas the Russian fox experiment was established using animals from an existing Canadian fox farm [8]. The farmed origin of these founders, and the pre-existence of domestication traits within them, forms a substantial component of Lord *et al*.'s [8] critique of the fox experiment, and domestication syndrome more generally. However, their review also provides a succinct history of fox farming, which reveals an obvious reason for why pre-domesticated foxes were used. In brief, early efforts at fox farming intensified as wild-caught pelts became rarer, but it proved extremely difficult to breed wild foxes in captivity [8,105]. All early attempts failed for the same reasons described in King & Donaldson's [102] rats; poor female fertility and maternal care were the primary hurdles. According to Lord *et al*. [8], ‘whether wild or captive born, most foxes would not breed in captivity, and females often ate their young'. A successful captive-breeding population was eventually established using large enclosures, rather than cages. Of note, relatively docile, less-stressed, female foxes appeared to out-reproduce their companions [8], with obvious implications for fitness and inheritance in later generations.

The similar traits documented in King & Donaldson's [102] rats and pre-experimental farmed foxes [8,76] reveal features of domestication syndrome in absence of experimental selection for tameness, thus suggesting other selective influences common to their caged captive environments. Statham *et al*. [106] discussed this prior selection in relation to the Russian foxes, describing their earlier farm history as a phase of ‘conscious' commercial selection for fur quality, along with ‘unconscious' behavioural selection for reproduction in caged captivity. This seems a reasonable depiction, but the presence of unconscious behavioural selection—occurring automatically as a result of captivity—complicates effects from later controlled experimental selection for tameness. Although domestication traits appear relatively enhanced in tamed experimental foxes [18,40,42], earlier unconscious selection for caged reproduction was sufficient to promote these traits in foxes [8,76], and in rats [102,103], suggesting a substantial confounding effect for any cage-based behavioural experiment. Other unconscious selective changes from the wild seem likely given reliable food provision and predator absence. The fact that domestication traits were also present in the ‘aggressive' lineage of Russian foxes [107]—those selected for the exact opposite of ‘tameness'—seems to confirm some confounding selective effect. As do findings of minimal cranial differences between tame and aggressive fox lineages when compared to their progenitor wild fox population [108].

Given these observations, any singular focus on experimental ‘selection for tameness' seems likely to obscure a wider range of other, largely unconscious, selective factors commonly experienced by wild populations transitioning to domestication. From this perspective, popular depictions of domestication syndrome as a suite of mysteriously associated traits and features that arise in response to ‘selection for tame behaviour' appear, at least, somewhat simplified, and potentially misleading. For example, assuming a singular selective catalyst for multiple and diverse traits of domestication logically leads to expectations of cryptic genetic mechanisms that could explain these trait associations; whereas, given a range of regularly shared selective changes affecting different aspects of domesticated phenotypes, as we propose (figure 1), such associative mechanisms would not be required.

## 8. Broader evolutionary implications

As noted, multiple previous authors have now highlighted traits and features resembling domestication syndrome in wild populations and taxa [29,47,51,52]. For example, Hare *et al*. [47] argue domestication-like differences between bonobos and chimpanzees result from wild bonobo ‘*self*-domestication'. They, and others (e.g. [109,110]), suggest enhanced female coalitionary behaviour in an isolated chimp-like ancestor altered sexual selection in this species by dampening the reproductive fitness of male aggression and dominance. Given this apparent example of wild domestication syndrome, Hare *et al*. [47] posited that self-domestication might occur in other wild populations, especially in island habitats (where relative docility is naturally common), and under close human presence. These speculations have since found support elsewhere—for example, in islandized rats [50], and in urban fox populations [52].

According to our selectively focused hypothesis, novel traits resembling domestication syndrome are entirely predictable wherever a given population experiences selective regime shifts similar to those seen in domestication. In effect, wild populations, or species, might experience effective self-domestication where ecological changes prompt selective shifts similar to those highlighted in figure 1. These seem especially likely in cases of novel islandization, where demographic or ecological difference could easily affect intra- or inter-sexual selection in males, or might shift predation and resource availability, or elevate maternal stress, promoting altered reproductive physiology in females. Several documented examples suggest such effects occurring. Sánchez-Villagra *et al*. [29] note that domestication-like traits in the Falkland Islands wolf and Balearian mouse-goat once prompted speculation of prehistoric human influence—since dismissed [111,112]—however, wild *self*-domestication in these species has not been extensively considered.

Intriguingly, multiple authors have discussed possible self-domestication in ancient hominins, including early *Homo sapiens* (e.g. [17,31,53–57,62,113–118]). There is debate over specific modes of selection [119], but, from our perspective, domestication traits in hominins imply reproductive regime shifts affecting male contest and female choice, or female reproductive physiology, or a combination of these factors. Inferable social dynamics seem relevant given past hominin evolution towards an obligate socio-cognitive niche with sophisticated social cooperation (e.g. [120–122]). Punitive egalitarian social mechanisms [57,123,124] and transition to ‘prestige-based' status competition [125] are both suggested to have dampened male intra-sexual dominance competition. In addition, longstanding alloparental support in hominins [126–130] implies maternal behavioural and physiological adaptations to highly social reproductive strategies, not seen in our extant Panin relatives.

## 9. Outstanding questions

We have outlined a selection-focused hypothesis of trait association under domestication syndrome, involving broad disruptions to sexually dimorphic reproductive regimes. This expanded focus upon multiple selective changes provides an inherently more complex explanatory hypothesis, likely to complicate future testing or validation attempts when compared to singular selection for tameness. Despite this, initial further research to explore this perspective could involve an expanded re-documentation of domestication literature, including observations where selection for tameness and pleiotropic genetic mechanisms are not overtly present. This work should consider the specific selective environments to determine whether, and to what extent, they entail predicted primary selective changes (figure 1); including altered male intra- and inter-sexual selection, or reproductively relevant female physiology, or both. Another potential avenue includes further review of existing island-mainland taxonomic comparisons for selective differences, and for symptoms resembling domestication syndrome. More general explorations might use phylogenetic comparisons of associated traits in taxa with overt forms of male sexual selection, resource abundance or elevated female stressors affecting some species, but not others.

As discussed, we argue shared disruption of reproductive regimes accounts for association of traits shared between different domesticated populations and taxa. However, previous findings have suggested correlation of some traits (e.g. colour and behaviour) within specific populations or lineages [39]—although, by contrast, others do not [131]. Where they do occur, these apparent associations do not preclude intra-population *selective* causes. However, our proposal also does not completely exclude genetic or biophysical forms of trait association. For example, Fallahshahroudi *et al*. [132] suggested altered pituitary function alone could explain several domestication-related traits in chickens. Similarly, androgens are recognized to influence both behaviour and morphology. By invoking a selective-level cause for shared domestication traits, our hypothesis can incorporate different biophysical avenues to effect similar trait changes. Closer examination of these associations, especially comparison of mechanisms driving similar trait changes in different taxa, may prove informative.

In closing, given that NCCs provide multiple widespread and diverse contributions to all vertebrate phenotypes, and that selective regimes differ dramatically between domesticated and wild contexts, evidence of selection on NCC genetics may simply reveal relatively standard evolutionary changes by various processes of selection, rather than supporting pleiotropic genetic association of domesticated traits. Like Johnsson *et al*. [6], we would like to see genetic research of domestication extend beyond publication of more genomic datasets showing evidence of selection affecting NCCs. We greatly appreciate the NCC hypothesis [1,5,64] and Russian fox experiment [18,40–42] for their scientific contributions, and for inspiring dramatically expanded interest in domestication and domestication syndrome. Overall, however, we hope our review and hypothetical proposal will enable researchers to better appreciate the potential complexity of domestication syndrome, and its wider implications for evolutionary theory.

## Data Availability

This article has no additional data.
